# Staged limbal stem cell transplantation and keratoplasty surgeries as a treatment for gelatinous drop-like corneal dystrophy

**DOI:** 10.3205/oc000210

**Published:** 2023-01-30

**Authors:** Tayaba N. Azher, Amanda C. Maltry, Joshua H. Hou

**Affiliations:** 1 Department of Ophthalmology and Visual Neurosciences, University of Minnesota, Minneapolis, United States

**Keywords:** gelatinous drop-like corneal dystrophy (GDLD), penetrating keratoplasty (PKP), allogeneic limbal stem-cell transplantation

## Abstract

Gelatinous drop-like corneal dystrophy (GDLD) is a rare autosomal recessive corneal dystrophy that has been associated with mutations in the TACSTD2 (M1S1) gene, which is normally expressed in corneal epithelial cells. GDLD is characterized by progressive deposition of amyloid in the corneal stroma with rapid recurrence in grafts after penetrating keratoplasty. We report of case of a patient with GDLD treated bilaterally with staged limbal stem cell transplantation and penetrating keratoplasty that resulted in long-term control of his disease. This case demonstrates that staged allogenic limbal stem cell transplantation, before or after penetrating keratoplasty, can be used to restore vision long-term in GDLD patients.

## Introduction

Gelatinous drop-like corneal dystrophy (GDLD), also known as primary familial amyloidosis or subepithelial amyloidosis, is a rare bilateral autosomal recessive corneal dystrophy presenting in the first two decades of life [1], [2]. GDLD was first described by Nakaizumi in 1914 and is found globally with increased prevalence in the Japanese population [1], [2], [3]. Clinically, patients present with foreign body sensation, severe photophobia, and vision loss [1]. GDLD is characterized by deposits of amyloid in the corneal subepithelial and stromal regions, which can progress to mulberry-like or gelatinous lesions on clinical exam [3].

Current treatment options, including superficial keratectomy, penetrating keratoplasty, lamellar keratoplasty, and penetrating limbo-keratoplasty [4], [5]. However, long-term outcomes are limited by recurrence of amyloid deposits in corneal grafts within a few years [6]. Use of keratoprosthesis has been described to achieve more stable long-term results, but recurrent amyloid deposition in the carrier cornea and peripheral host cornea can lead to persistent photophobia and foreign body sensation [7]. As such, therapies to treat the underlying disease in GDLD have been limited to date. 

Allogeneic limbal stem-cell transplantation to replace the host limbus has a been described as a promising therapeutic option in GDLD due to the fact that GDLD is associated with mutations in the TACSTD2 (M1S1) gene, a gene normally expressed in corneal epithelial cells [8], [9], [10]. Both Shimazaki et al. and Movahedan et al. reported good outcomes with at least 9 months of follow-up with combined allogeneic limbal stem cell transplantation and keratoplasty (lamellar or penetrating) for treatment of GDLD [6], [11]. However, in all reported cases, limbal stem cell transplantation was performed simultaneously with keratoplasty. We report two cases where allogeneic limbal stem cell transplantation was performed before and after penetrating keratoplasty, respectively, for the treatment of GDLD. Our cases help demonstrate that limbal stem cell transplantation and keratoplasty can be performed in a staged manner and still be valuable in restoring vision in patients with gelatinous drop-like corneal dystrophy. 

## Case description

A 34-year-old Somali male presented initially with pain, photophobia, and blurry vision. He had a history of consanguineous parents and three siblings with similar vision problems. His past ocular history was notable for two previous penetrating keratoplasties (PKP) in his right eye and one in his left eye. On exam, his vision was light perception in both eyes with right eye esotropia. On exam under anesthesia, slit lamp exam showed diffuse corneal scars and vascularization over both corneas. Mulberry-like anterior stromal deposits were noted. Intraocular pressure and B-scan were normal in both eyes.

**Figure 1:**
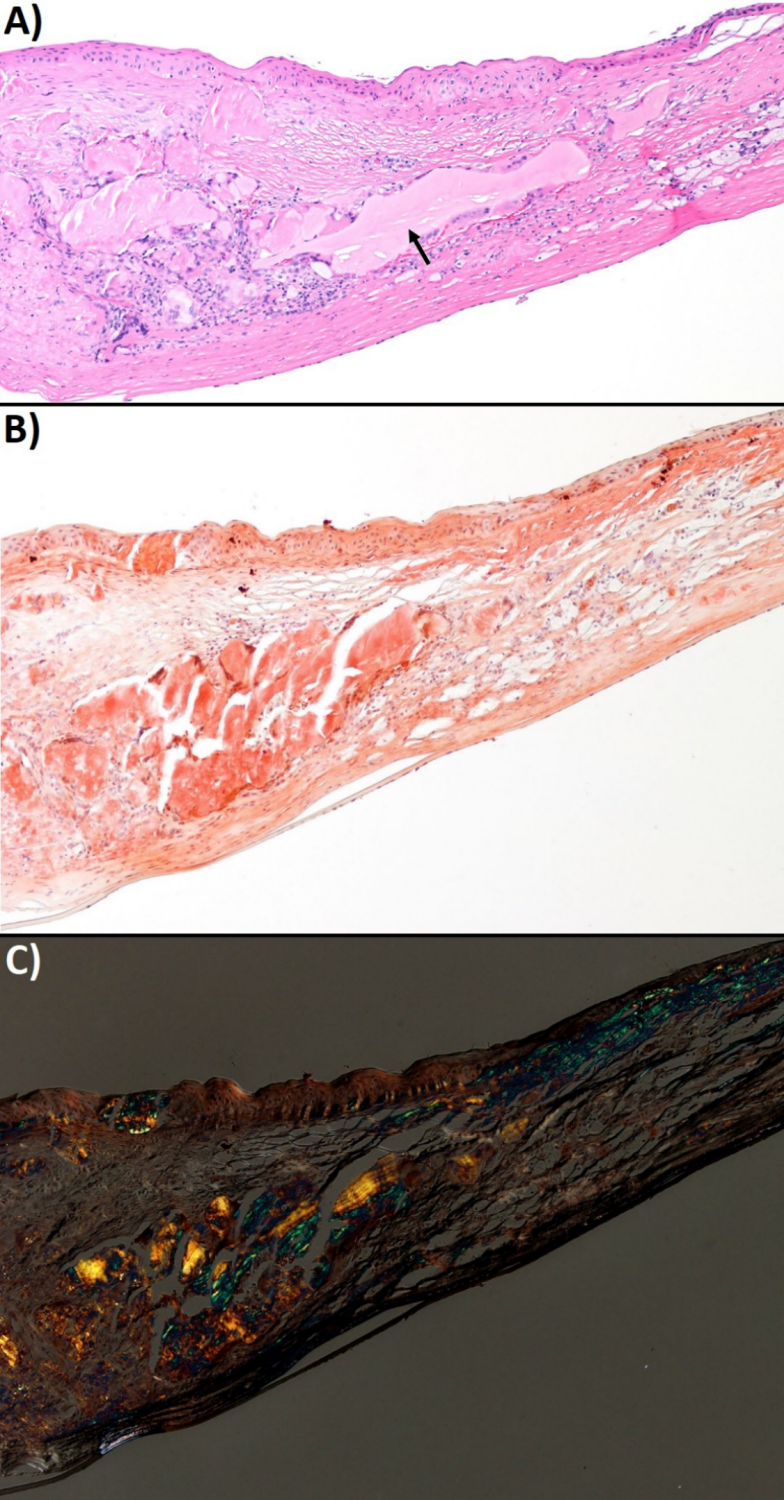
Histopathology of the recipient’s left cornea. Amyloid is noted in the corneal stroma on hematoxylin and eosin stain (A), congo red stain (B), congo red birefringence (C).

GDLD was suspected; however, in order to confirm the diagnosis, a repeat PKP was performed in the left eye with the patient’s cornea sent to pathology. A diagnosis of stromal amyloidosis consistent with GDLD was subsequently confirmed on histology (Figure 1 (Fig. 1)). One year post-operatively, early signs of recurrence of GDLD were noted with peripheral sub-epithelial irregularity. Deposition of subepithelial amyloid continued to worsen until 3 years post-PKP, when keratolimbal allografting (KLAL) plus superficial keratectomy was performed to salvage the cornea and prevent further stromal deposition of amyloid. Following the procedure, the patient was started on oral mycophenolate 750 mg twice a day, tacrolimus 2 mg twice a day, and prednisone 60 mg daily to prevent graft rejection. Prophylactic oral valaciclovir and trimethoprim/sulfamethoxazole were also started per standard immunosuppression protocols. Topical artificial tears, Ofloxacin, and Prednisolone acetate were also started post-operatively. One month after surgery, the patient’s VA was 20/100 in the left eye and the PKP graft was clear without deposits.

**Figure 2:**
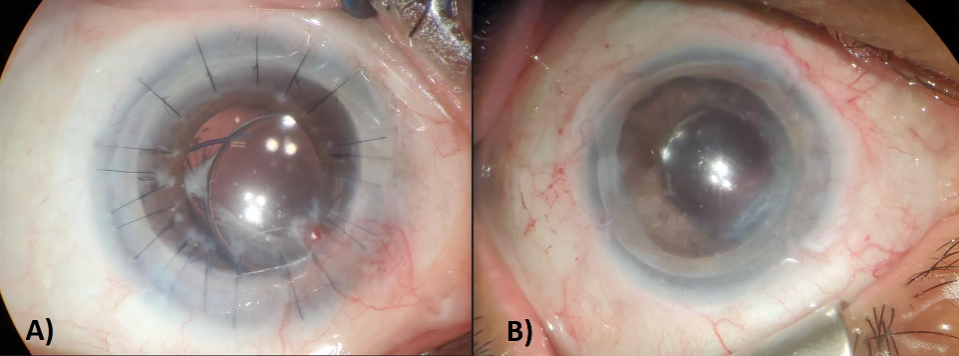
Post-surgical photograph of the (A) right cornea and limbus 2 years after keratolimbal allograft (KLAL) and 1.5 years after penetrating keratoplasty (PKP); and the (B) left cornea and limbus 5 years after PKP and 3 years after KLAL. The PKP graft remained clear in both eyes.

The patient then underwent KLAL in the right eye. Four months after KLAL, the patient continued to have pain, photophobia, and blurry vision in the right eye due to persistent anterior corneal stromal mulberry-like amyloid deposits. The patient then underwent PKP with cataract extraction without complications. The patient’s left cornea was sent to lab and histopathology again confirmed stroma amyloid consistent with GDLD. At the last follow-up 2 years after KLAL in the right eye and 3 years after KLAL in the left eye, both PKP grafts were clear (Figure 2 (Fig. 2)). Final uncorrected visual acuity was count fingers in the right eye and 20/70 in the left eye. The limited vision in the right eye was believed to be due to long-standing depravational amblyopia consistent with his right eye esotropia. 

## Discussion

Gelatinous drop-like corneal dystrophy is a rare autosomal recessive disorder affecting the corneal epithelium and anterior corneal stroma. Deposition of subepithelial and anterior stromal amyloid results in corneal opacification and decreased vision in the first two decades of life. Current therapeutic options include superficial keratectomy, penetrating keratoplasty, lamellar keratoplasty, and penetrating limbo-keratoplasty but long-term outcomes with these procedures are limited [5], [6]. Lesions rapidly reoccur in new grafts requiring repeat surgeries every few years. 

Limbal-stem cell transplantation has been proposed to treat the underlying pathology associated with GDLD; however, to date, only cases of simultaneous PKP and KLAL have been reported [6], [11]. In some patients, a PKP performed prior to KLAL may be important for histological confirmation of the diagnosis prior to committing a patient to the systemic immunosuppression required to maintain a limbal transplant. Additionally, evidence suggests that PKP done simultaneously with KLAL may have worse outcomes than staged KLAL followed by PKP due to increase rates of KLAL graft rejection with simultaneous surgery [12]. As such, performing KLAL prior to PKP or PKP prior to KLAL may be preferable for some patients depending on the situation.

In our patient, the limbal stem cell transplantation and PKP surgery were separated in time in both eyes with good outcomes out to 5 years. Our patient underwent PKP followed by KLAL 3 years after initially transplantation in the left eye. Despite early recurrence of subepithelial amyloid deposits within 1 year after PKP alone, KLAL with superficial scrapping was sufficient to salvage the graft even 3 years out. This suggests that performing PKP to confirm diagnosis can be safely performed with a significant time lag until KLAL must be performed. 

In his right eye, the patient underwent limbal stem cell transplantation first followed by PKP four months later. Although final visual outcome was limited by presumed deprivation amblyopia, the graft clarity was maintained without recurrent amyloid deposits out to 3 years. Though the patient continued to have blurred vision and photophobia, normal corneal epithelium over a mulberry-like diseased cornea was achievable with KLAL alone. Subsequent PKP was effective in reducing the patient’s photophobia and pain long-term.

## Conclusion

To the best of our knowledge, our case is first to report staged KLAL and PKP for treatment of GDLD. This can be an excellent option for patients for long-term visual rehabilitation. PKP followed by KLAL allows for pathologic diagnosis of disease prior to performing KLAL. In such cases, KLAL can be performed up to 3 years after the PKP without compromising the long-term clarity of the graft. KLAL followed by PKP can also be performed and may have the advantage of better long-term outcomes for the KLAL graft. In such cases, a stable ocular surface is achievable with KLAL alone, even with persistent amyloid deposits in the cornea stroma.

## Notes

### Competing interests

The authors declare that they have no competing interests.
